# Fibrinogen is not elevated in the cerebrospinal fluid of patients with multiple sclerosis

**DOI:** 10.1186/2045-8118-8-25

**Published:** 2011-10-26

**Authors:** Rainer Ehling, Franziska Di Pauli, Peter Lackner, Bettina Kuenz, Wolfram Santner, Andreas Lutterotti, Claudia Gneiss, Harald Hegen, Michael Schocke, Florian Deisenhammer, Thomas Berger, Markus Reindl

**Affiliations:** 1 Clinical Department of Neurology, Innsbruck Medical University, Anichstrasse 35, 6020 Innsbruck, Austria; 2 Department of Radiology, Innsbruck Medical University, Anichstrasse 35, 6020 Innsbruck, Austria

**Keywords:** blood-CSF barrier (BCSFB), cerebrospinal fluid (CSF), central nervous system (CNS), fibrinogen, neuroinflammation

## Abstract

**Background:**

Elevated plasma fibrinogen levels are a well known finding in acute infectious diseases, acute stroke and myocardial infarction. However its role in the cerebrospinal fluid (CSF) of acute and chronic central (CNS) and peripheral nervous system (PNS) diseases is unclear.

**Findings:**

We analyzed CSF and plasma fibrinogen levels together with routine parameters in patients with multiple sclerosis (MS), acute inflammatory diseases of the CNS (bacterial and viral meningoencephalitis, BM and VM) and PNS (Guillain-Barré syndrome; GBS), as well as in non-inflammatory neurological controls (OND) in a total of 103 patients. Additionally, MS patients underwent cerebral MRI scans at time of lumbar puncture.

CSF and plasma fibrinogen levels were significantly lower in patients with MS and OND patients as compared to patients with BM, VM and GBS. There was a close correlation between fibrinogen levels and albumin quotient (rho = 0.769, *p *< 0.001) which strongly suggests passive transfer of fibrinogen through the blood-CSF-barrier during acute inflammation. Hence, in MS, the prototype of chronic neuroinflammation, CSF fibrinogen levels were not elevated and could not be correlated to clinical and neuroradiological outcome parameters.

**Conclusions:**

Although previous work has shown clear evidence of the involvement of fibrinogen in MS pathogenesis, this is not accompanied by increased fibrinogen in the CSF compartment.

## Background

Fibrinogen is a soluble 340 kDa dimeric glycoprotein that is synthesized in the liver, secreted into the plasma and able to signal via a number of receptors expressed on cells of the hematopoietic, immune and nervous systems [1,2]. Apart from its pivotal role in thrombogenesis, inflammation, immune responses and atherogenesis, it is also a prominent acute-phase reactant. Transiently elevated plasma fibrinogen levels have been described in acute infectious diseases, in acute stroke and myocardial infarction; chronically raised plasma fibrinogen levels have been associated with an increased risk for cardiovascular diseases [3]. Whilst the significance of plasma fibrinogen is well established, the determination of fibrinogen in cerebrospinal fluid (CSF) has so far been restricted to descriptions of elevated fibrinogen degradation products in the CSF of patients with subarachnoid haemorrhage, traumatic brain injury and Guillain-Barré syndrome (GBS) [4-6].

Accumulations of fibrinogen and its degradation products have been demonstrated in the central nervous system (CNS) tissue of stroke, bacterial meningitis (BM), HIV-encephalitis, Alzheimer's disease and multiple sclerosis (MS) patients [7]. In MS, the leakage of fibrinogen into the CNS was found to result in microglial activation and plaque formation [8]. In experimental allergic encephalomyelitis (EAE), an animal model of MS, a close correlation was found between perivascular fibrin deposition and relapses and, moreover, pathology was ameliorated after administration of anti-coagulants or fibrinogen depletion [9,10].

The aim of this study was to determine the value of fibrinogen as biomarker in plasma and CSF of patients with MS compared to other neurological diseases, and to analyze potential clinical and neuroradiological correlations.

## Methods

### Patients

A total of 103 patients were recruited between 2004 and 2009 at the Clinical Department of Neurology, Innsbruck Medical University, Austria, with approval of the local ethical committee (study Nr. UN2045, 217/4.12). All patients gave written informed consent and were assigned to the following groups:

1. Clinically definite MS patients (n = 40), 27 with relapsing-remitting (RR) and 13 with chronic progressive (CP) disease course. Acute relapse was present at time of CSF analysis in 30% of MS patients. All MS patients were examined by a standardized brain MRI protocol including Gadolinium-DTPA and scanned using a 1.5 T MR scanner (Magnetom Avanto, Siemens, Germany) at the time of diagnostic lumbar puncture, again prior to the application of methylprednisolone.

2. Patients with BM (n = 15).

3. Patients with viral meningoencephalitis (VM, n = 14). These two groups (2 and 3) were included as controls, reflecting an acute inflammatory CNS disease. The neurological outcome of these patients was evaluated at discharge from clinics by chart review using the Glasgow Outcome Scale (GOS) [11,12].

4. Patients with GBS (n = 14) comprised a group with acute inflammatory disease of the peripheral nervous system (PNS). All GBS patients were assessed using standard nerve conduction studies within 6.2 ± 7.7 (mean ± STD) days after disease onset and classified according to common criteria [13] into demyelinating (60%), axonal (26.7%) and normal (13.3%). Disabilities at nadir were scored from 1-5 according to the Hughes Functional Grading Scale (HGS) [14].

5. Patients with other, non-inflammatory neurological diseases (OND, n = 20) consisted of patients with chronic back pain (n = 6), chronic headache (n = 5), ischemic transverse myelopathy (n = 4) and Bell's palsy (n = 5). To exclude any biasing inflammatory conditions, patients with OND were only included if they had a normal CSF evaluation.

### Sample collection and analysis

Plasma, serum and CSF samples were obtained during standard diagnostic lumbar and peripheral vein puncture, and immediately analyzed for CSF white cell count (WCC), CSF/serum glucose ratio (GluR), CSF/serum albumin quotient (Qalb), IgG index, total protein levels, plasma fibrinogen and serum C-reactive protein (CRP) levels using standard methods: photometric measurement for GluR and total CSF protein, nephelometric measurement for serum and CSF IgG, ELISA for plasma fibrinogen and immunturbidimetric measurement for CRP [15]. CSF fibrinogen was analyzed at a dilution of 1:51 using commercially available ELISA according to manufacturer's protocol (RK024A, Hyphen Biomed, France). Fibrinogen quotient was calculated using the formula: CSF fibrinogen/plasma fibrinogen. To discriminate between a passive transfer of fibrinogen through the blood-CSF barrier (BCSFB) and intrathecal fibrinogen production, we calculated an index using the formula: (CSF fibrinogen/plasma fibrinogen)/Qalb. CSF specimens exceeding a red blood cell count of 33/μl were excluded to avoid fibrinogen contamination due to fibrinolysis. All samples were collected prior to the application of anti-infective, corticosteroid or other immunomodulatory or immunosuppressive treatment.

### Data analysis

Patient characteristics were compared between diagnostic groups by Kruskal-Wallis test or chi-square test when appropriate. Spearman's rho was calculated and rho > 0.4 was considered as a relevant correlation. GOS at hospital discharge of patients with BM and VM were grouped into favorable (GOS = 5) and unfavorable outcome (GOS < 5). Disabilities of GBS patients at the nadir were scored according to the HGS and dichotomized into two groups (< 4 and > = 4). CSF data were compared between groups by Kruskal-Wallis test, post-hoc analysis was done by Dunn's Multiple Comparison Test. Calculations were done using PASW 18 (IBM, Chicago, IL, USA), and GraphPad Prism 5.00 (GraphPad Software, San Diego, CA, USA).

## Results

Groups differed significantly in age and sex with an older age and female predominance in the GBS group (Table 1). As expected, patients with acute inflammatory disease of the CNS (BM and VM) had significantly elevated CSF WCC and elevated protein levels. Additionally, the group with BM had a significantly reduced GluR. The MS cohort had pathologically raised intrathecal immunoglobulin production of the IgG subtype and 97.5% had CSF-specific oligoclonal IgG bands (Table 1). Cerebral MRI showed significantly more contrast enhancing lesions (CEL) in RR-MS patients as compared to patients with CP-MS (2.6 ± 6.2 versus 0.5 ± 0.8, number of CEL, mean ± STD, *p *< 0.001), but significantly less T2 hyperintense lesions (26.9 ± 25.9 versus 46.5 ± 27.3, number of T2 hyperintense lesions, mean ± STD, *p *< 0.001).

**Table 1:** Characteristics for patients in the five groups studied

	BM	VM	MS	GBS	OND	p-value
n	15	14	40	14	20	
mean age in years (STD)	46.2 (17.0)	41.7 (16.3)	41.2 (11.4)	60.3 (18.1)	39.9 (12.5)	0.012
females (%)	5 (33.3)	6 (42.9)	27 (67.5)	2 (14.3)	11 (55.0)	0.007
CSF WCC/μl (mean, STD)	1612.3 (2042.0)	170.8 (124.0)	9.0 (9.1)	6.1 (6.4)	1.9 (1.7)	< 0.001
IgG-index (mean, STD)	0.7 (0.1)	0.5 (0.1)	1.2 (0.7)	0.5 (0.1)	0.5 (0.2)	< 0.001
oligoclonal IgG bands (%)	nd	nd	97.5	nd	nd	
Qalb (mean, STD)	50.8 (40.8)	17.7 (8.5)	5.6 (2.5)	13.1 (9.2)	6.7 (3.6)	< 0.001
CSF/serum glucose ratio (mean, STD)	0.3 (0.2)	0.7 (0.2)	0.7 (0.2)	0.8 (0.4)	0.7 (0.1)	< 0.001
protein (mg/dl, mean, STD)	354.9 (383.8)	116.2 (44.3)	43.0 (15.1)	76.1 (51.7)	38.4 (10.7)	< 0.001
CRP serum (mg/dl, mean, STD)	12.5 (11.2)	0.9 (1.4)	0.3 (0.7)	2.0 (3.8)	0.4 (0.5)	< 0.001

BM, bacterial meningitis; VM, viral meningoencephalitis; MS, multiple sclerosis; GBS, Guillain-Barré syndrome; OND, other non-inflammatory neurological diseases; STD, standard deviation; CSF, cerebrospinal fluid; WCC, white cell count; Qalb, CSF/serum albumin quotient.

**Figure 1:**
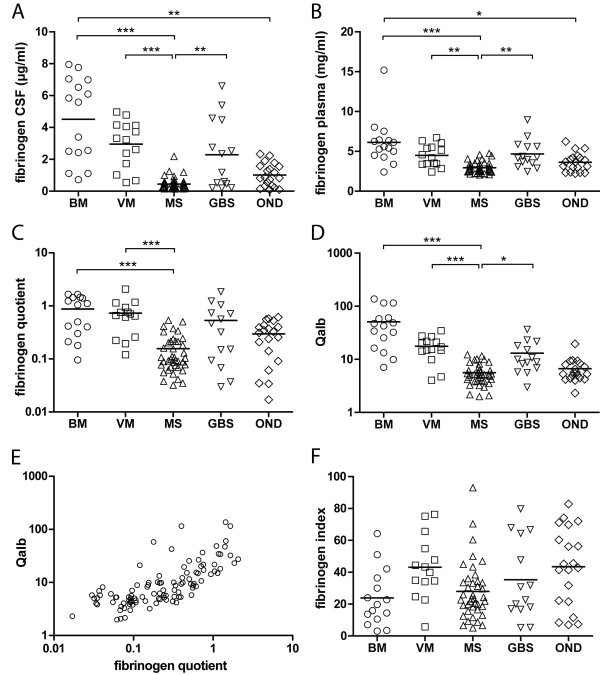
**Data plots of fibrinogen concentration in the five investigated patient groups**. A: fibrinogen in CSF, B: fibrinogen in plasma. MS patients had significantly lower fibrinogen levels in CSF and plasma as compared to patients with BM, VM and GBS. In addition, OND patients had lower fibrinogen levels in CSF and plasma as compared to patients with BM (bar = mean, * *p *< 0.05, ** *p *< 0.01, *** *p *< 0.001). C: Data plots of fibrinogen quotient (CSF/serum) for the five patient groups, showing significantly lower values in patients with MS as compared to patients with BM and VM (bar = mean, *** *p *< 0.001). D: Data plots of albumin quotient (Qalb) for the five patient groups. Patients suffering from MS had a significantly lower Qalb compared to those with acute inflammatory diseases of the CNS (BM and VM) reflecting an altered BCSFB status. Qalb was also significantly lower in patients with MS as compared to patients with GBS, reflecting, in this context, an altered brain-nerve barrier status in GBS. However, Qalb did not differ between MS and OND patients (bar = mean, * *p *< 0.05, *** *p *< 0.001). E: Scatter plot of albumin quotient against fibrinogen quotient for all patients. Qalb was significantly correlated to fibrinogen quotient (rho = 0.769, *p *< 0.001). F: Data plot of the fibrinogen index [(CSF fibrinogen/plasma fibrinogen)/Qalb] for the five patient groups. The overall statistical significance between groups was *p *< 0.012; at the group level there was a non-significant trend towards lower values in patients with BM as compared to patients with VM and patients with OND.

MS patients had significantly lower fibrinogen levels in CSF and plasma compared to patients with BM (both *p *< 0.001), VM (*p *< 0.001, *p *< 0.01) and GBS (both *p *< 0.01). Additionally, OND patients had lower fibrinogen levels in CSF and plasma compared to patients with BM (*p *< 0.01; *p *< 0.05, respectively; Figure 1A and 1B).

The fibrinogen quotient in MS patients was significantly lower than in patients with BM and VM (both *p *< 0.001; Figure 1C). Qalb was significantly lower in MS than in BM or VM (both *p *< 0.001) and also lower than in GBS (*p *< 0.05), whereas there was no difference to OND (Figure 1D). Using data for all patients, Qalb was significantly correlated to the fibrinogen quotient (rho = 0.769, *p *< 0.001, Figure 1E). The fibrinogen index, which was calculated in order to discriminate between a passive transfer of fibrinogen through the BCSFB and intrathecal production of fibrinogen, showed a non-significant trend towards a lower index in patients with BM when compared to patients with VM and patients with OND. However, the overall statistical significance between groups was *p *< 0.012 (Figure 1F).

Significant correlations were found between serum CRP and CSF fibrinogen (rho = 0.483, *p *< 0.001) and plasma fibrinogen (rho = 0.738, *p *< 0.001; data not shown). Fibrinogen was independent of age for all investigated groups. Fibrinogen levels did not differ for different clinical disease courses or during acute relapses in MS patients when compared to times of remission or chronic progression. Furthermore, CSF, plasma fibrinogen and fibrinogen quotient were independent of the number of T2-hyperintense lesions and the number of CEL on cerebral MRI scans. Patients with BM and VM did not differ in clinical outcome using GOS with respect to their fibrinogen levels. In the GBS group, the fibrinogen status was independent of the electrophysiological form of the disease and the severity of the disease at nadir (data not shown).

## Discussion

This study was designed to investigate a possible involvement of CSF fibrinogen in MS pathogenesis during times of acute inflammation, since vascular abnormalities and accumulation of perivascular fibrin are key findings in MS [8]. Thus, the finding of normal fibrinogen values in CSF and plasma of MS patients in our study was unexpected. A possible explanation for this observation could be a restriction of blood-brain barrier (BBB) breakdown to local areas of acute inflammatory demyelination as opposed to a diffuse and widespread BBB breakdown in infectious meningoencephalitis. Although BBB breakdown in active MS plaques enables the deposition of fibrinogen, it is not necessarily associated with a more permeable BCSFB, thereby preventing a detectable increase of fibrinogen in the CSF compartment [16]. Restricted and focal damage of the BBB in MS may also account for the absence of elevated CSF fibrinogen levels during acute relapses and the lack of correlation to CEL. The high molecular weight of fibrinogen as compared to the lower molecular weight marker gadolinium used for imaging studies may be an additional negative confounder.

However, our study found highly increased fibrinogen levels in the acute inflammatory conditions that are associated with a diffuse alteration in the state of the BCSFB, where it is a highly sensitive marker for measuring acute neuroinflammation. The subsequent altering of the extracellular matrix of the brain parenchyma by fibrinogen has been shown to inhibit neuroregeneration and potentiate inflammation in experimental animal models [9,17]. The finding of elevated CSF fibrinogen holds not only true for the CNS, but also for an acute inflammatory condition of the PNS. The finding of increased CSF fibrinogen levels in GBS is in line with previous studies [6] and of particular interest since the impairment of the blood-nerve barrier in this context is irrespective of an infectious agent.

Due to the relatively small number of patients investigated and the retrospective design, our study was not powered to detect other influencing factors on the plasma fibrinogen status [3] and the conclusions may therefore be limited. However, our study has demonstrated elevated levels of CSF fibrinogen in patients with acute inflammatory diseases of the CNS and the PNS, where to our best knowledge influencing factors have not been thoroughly investigated. In these acute inflammatory disorders fibrinogen may enter the CSF directly through the BCSFB or indirectly through the BBB and make an important contribution to inflammatory and regenerative processes. In contrast, CSF fibrinogen is not increased in MS patients, where an apparently intact BCSFB prevents fibrinogen entering the CSF. This feature may be useful to distinguish MS from acute CNS inflammatory diseases.

## List of abbreviations

BBB: blood-brain barrier; BCSFB: blood-CSF barrier; BM: bacterial meningoencephalitis; CEL: contrast enhancing lesions; CSF: cerebrospinal fluid; CNS: central nervous system; CP: chronic progressive; EAE: experimental allergic encephalomyelitis; GBS: Guillain-Barré syndrome; GluR: CSF/serum glucose ratio; GOS: Glascow Outcome Scale; HGS: Hughes Functional Grading Scale; MS: multiple sclerosis; OND: other neurological controls; PNS: peripheral nervous system; Qalb: CSF/serum albumin quotient; RR: relapsing-remitting; VM: viral meningitis; WCC: white cell count.

## Competing interests

The authors declare that they have no competing interests.

## Authors' contributions

Conceived and designed the study: RE BK MR. Patient recruitment: RE BK FdP PL AL CG HH TB. Analyzed the data: RE PL WS MS FD MR. Wrote the paper: RE MR. All authors read and approved the final manuscript.
